# Gender, aging and longevity in humans: an update of an intriguing/neglected scenario paving the way to a gender-specific medicine

**DOI:** 10.1042/CS20160004

**Published:** 2016-08-23

**Authors:** Rita Ostan, Daniela Monti, Paola Gueresi, Mauro Bussolotto, Claudio Franceschi, Giovannella Baggio

**Affiliations:** * Interdepartmental Centre “L. Galvani” (CIG) and Department of Experimental, Diagnostic and Specialty Medicine (DIMES), University of Bologna, Via San Giacomo 12, 40126 Bologna, Italy; † Department of Clinical and Experimental Biomedical Sciences, University of Florence, Viale Morgagni 50, 50134 Florence, Italy; ‡ Department of Statistical Sciences “Paolo Fortunati”, University of Bologna, Via Belle Arti 41, 40126 Bologna; § Internal Medicine Unit, Department of Molecular Medicine, University of Padua, Italy; ║ IRCCS, Institute of Neurological Sciences of Bologna, 40139 Bologna, Italy

**Keywords:** aging, centenarians, gender, gender-specific medicine, gut microbiome, longevity, mitochondrial DNA, X chromosome inactivation

## Abstract

Data showing a remarkable gender difference in life expectancy and mortality, including survival to extreme age, are reviewed starting from clinical and demographic data and stressing the importance of a comprehensive historical perspective and a gene–environment/lifestyle interaction. Gender difference regarding prevalence and incidence of the most important age-related diseases, such as cardiovascular and neurodegenerative diseases, cancer, Type 2 diabetes, disability, autoimmunity and infections, are reviewed and updated with particular attention to the role of the immune system and immunosenescence. On the whole, gender differences appear to be pervasive and still poorly considered and investigated despite their biomedical relevance. The basic biological mechanisms responsible for gender differences in aging and longevity are quite complex and still poorly understood. The present review focuses on centenarians and their offspring as a model of healthy aging and summarizes available knowledge on three basic biological phenomena, i.e. age-related X chromosome inactivation skewing, gut microbiome changes and maternally inherited mitochondrial DNA genetic variants. In conclusion, an appropriate gender-specific medicine approach is urgently needed and should be systematically pursued in studies on healthy aging, longevity and age-related diseases, in a globalized world characterized by great gender differences which have a high impact on health and diseases.

## INTRODUCTION

Lifespan and longevity are complex and multifactorial traits resulting from an intriguing combination of ‘Nature’ and ‘nurture’, the unique reciprocal interaction between environmental, genetic, epigenetic and stochastic factors, each contributing to the overall phenotype [1,2].

Women live longer than men and this difference in life expectancy is a worldwide phenomenon indicating that human longevity seems strongly influenced by gender defined as the combination between biological sexual characteristics (anatomy, reproductive functions, sex hormones, expression of genes on the X or Y chromosome) and factors related to behaviour, social role, lifestyle and life experiences [3–6].

Following a historical perspective, in Europe in the 19th Century, life expectancy was less than 40 years and longevity of the two genders was generally very similar. The high female mortality due to pregnancy and childbirth corresponded to a higher male mortality from causes related to work, accidental injury or violence. Moreover, infectious and communicable diseases affected and killed men and women almost equally [7]. Throughout the 20th Century, mortality became concentrated in the older ages, non-communicable diseases became the prevailing causes of death, and a female survival advantage emerged and grew. This divergence in life expectancy can partly be explained by the declining rates in maternal mortality; however, a major contribution is due to differences in behaviour and biology between males and females [8].

Using historical data from 1763 birth cohorts from 1800 to 1935 in 13 developed countries, Beltrán-Sánchez et al. [9] showed that gender asymmetry emerged in cohorts born after 1880, that excess adult male mortality is rooted in a specific age group (50–70) and that heart disease is the main condition associated with increased excess male mortality in birth cohorts of 1900–1935. The authors have suggested that excess male mortality, found even after accounting for smoking-attributable deaths, may be explained by underlying traits of vulnerability to CVD (cardiovascular disease) that emerged with the reduction of infections and changes in diet and other lifestyle factors [9].

The maximum difference in life expectancy between males and females was found between the 1970s and the 1990s. The subsequent reduction of the gender gap can be attributed partly to the narrowing of differences in risk behaviours between males and females, along with the decline in mortality rates from CVD among men [10]. In the EU-28 countries, the difference in life expectancy between males and females was 5.5 years in 2013 (http://ec.europa.eu/eurostat/statistics-explained/index.php/Mortality_and_life_expectancy_statistics); however, the gender gap varied largely across EU member states.

The survival advantage of women is counterbalanced by a worse quality of life in advanced age due to the increase in disability and degenerative diseases [12]. Therefore men and women have a diverse chance to attain longevity and, at the same time, the aging process is qualitatively different between genders.

The impact of gender difference in aging has been extensively assessed, but the study of the interaction between a series of fundamental aspects such as hormonal, immunological and metabolic pathways as well as genetic background remains largely unknown.

Accordingly, the present review aims to (i) give an accurate analysis of mortality causes and age-related diseases pattern in men and women; (ii) describe the most important mechanisms underpinning the gender difference in longevity and aging (sex hormones, immunity, genetic factors, nutrition and stress); (iii) attempt to explain the difference in longevity between males and females, in human models of extreme longevity such as centenarians and long-lived families, suggesting the importance of an integrated investigation of nuclear, mitochondrial DNA genetics and gut microbiome; and (iv) stress the urgent need for a gender-specific medicine, taking into account the profound differences in pathophysiological pathways, in clinical characteristics and in pharmacological response between men and women. In conclusion, the scientific world is obliged to revise all outcomes in all fields of medicine on the basis of gender differences.

## GENDER AND AGE-RELATED DISEASES

The epidemiology of age-related diseases is substantially different between genders and changes dramatically in women after menopause [13]. Table 1 reports mortality data by sex regarding the 14 leading causes of death in U.S.A. in 2013 and refers to all races and ages [14]. Women died at higher rates than men of chronic lower respiratory diseases, cerebrovascular diseases, AD (Alzheimer's disease), influenza and pneumonia, septicaemia and hypertension-related diseases [14]. Even in the EU, a significant gender gap exists in mortality rates in all countries. In particular, death rates for IHD (ischaemic heart disease) and stroke are higher for men than for women [10].

**Table 1:** Number of deaths, percentage of total deaths by sex regarding the 14 leading causes of death in U.S.A. in 2013 Data refer to all races and all ages. Adapted from [14].

		Male		Female		
Rank	Cause of death	Number	Total deaths (%)	Number	Total deaths (%)	Higher mortality for
1	Heart disease	321,347	24.6	289,758	22.4	♂
2	Malignant neoplasms	307,559	23.5	277,322	21.5	♂
3	Chronic lower respiratory diseases	70,317	5.4	78,888	6.1	♀
4	Accidents (unintentional injuries)	81,916	6.3	48,641	3.8	♂
5	Cerebrovascular diseases	63,691	4.1	75,287	5.8	♀
6	Alzheimer's disease	25,836	2.0	58,931	4.6	♀
7	Diabetes mellitus	39,841	3.1	35,737	2.8	♂
8	Influenza and pneumonia	26,804	2.1	30,175	2.3	♀
9	Kidney diseases	23,493	1.8	23,619	1.8	=
10	Suicide	32,055	2.5	9,094	0.7	♂
11	Septicaemia	17,994	1.4	20,162	1.6	♀
12	Chronic liver disease and cirrhosis	23,709	1.8	12,718	1.0	♂
13	Essential hypertension-related diseases	12,963	1.0	17,807	1.4	♀
14	Parkinson's disease	15,088	1.2	10,108	0.8	♂
15	All other causes	253,421	19.4	302,707	23.5	♂

There are important inequalities in healthy life years between men and women. In EU countries, life expectancy at age 50 reached 29.8 years for men and 34.6 years for women in 2010, but the average duration of life free from activity limitation remained practically the same in women (68.6 years) and men (67.9 years) [10,15,16], meaning that the almost 5 years of advantage in life expectancy of women are years of diseases and disability.

### Cardiovascular disease (CVD)

Differences between women and men in the epidemiology, pathophysiology and symptoms of CVD are well-described. This gender gap should been taken into account because it strongly impinges on the effects of specific drugs and outcomes. Both factors linked to sex (gene expression from the sex chromosomes, sex hormones, metabolism of drugs by sex-specific cytochrome expression) and gender (sociocultural processes, behaviours, exposure to specific environment, nutrition, lifestyle and attitudes towards treatments and prevention) play a fundamental role in determining CVD risk [17].

Death rates for IHD are 70% higher for men than for women on average in all EU countries [10]. In addition, women showed a delayed onset of IHD (7–10 years on average) in several western EU states, even though, due to harmful lifestyle modifications, the prevalence of IHD is increasing in young women [17]. Moreover, IHD in women may show different symptoms and pain localization, and may need diverse diagnostic procedures and drugs [18].

HF (heart failure) is one of the major health threats of Western societies and affects up to 10% of the elderly, in absolute numbers more women than men [19]. However, women survive better than men and HF in women frequently occurs at older age and with less ischaemic aetiology than in men [20]. Recent data from the Framingham Heart Study showed that, in the latter half of the 20th Century, incidence of HF has declined by about one-third in women, but not in men, even though, after adjusting for age, survival after HF onset was improved in both genders [21].

The difference in the epidemiology of hypertension between men and women deeply changes with age. In particular, hypertension has a low prevalence in young and adult age when it is more predominant among men. By contrast, hypertension is more common in women than in men in the elderly population [17]. Indeed, falling oestrogen production during and after menopause has been associated with hypertension in women [22].

During aging, there is a sex-specific ‘cardiac remodelling’. In particular, women develop more frequently concentric cardiac hypertrophy with smaller internal cavity and relatively larger wall thickness, preserving a better ejection fraction and myocardial contractility than men. On the other hand, men show more frequently eccentric hypertrophy leading to an increased stroke volume and dilatation [17].

Women are particularly susceptible to the deleterious impact of T2D (Type 2 diabetes) and hypertension on cardiovascular health. These conditions were associated with higher risk of HF in women with respect to men (T2D increases 3.4-fold and 2-fold the risk of HF in women and men respectively; hypertension increases 5-fold and 2-fold the risk of HF in women and men respectively) [21]. In addition, T2D worsens the coronary artery disease outcome more in women than in men [23]. Finally, some pregnancy-associated conditions, such as pre-eclampsia and other hypertensive disorders, further contribute to increased risk for future chronic hypertension, CVD, cerebrovascular diseases and death in women [24].

### Type 2 diabetes (T2D)

Recent data show that the difference in the global estimates of T2D between men and women in terms of cases (male, 197.7 million; female, 184.1 million), prevalence (male, 8.7%; female, 8.1%) and age-specific prevalence [25] is small. Even if T2D prevalence is similar in men and women, it is slightly higher in men under 60 years of age and in women at older ages. Indeed, the longer survival of women is one of the factors leading to a higher prevalence of diabetes for women than for men at advanced age [26]. However, a stronger connection between diabetes and coronary heart disease has been demonstrated in women. In particular, the relative risk for mortality due to coronary heart disease in diabetic patients is 50% higher in women than in men, suggesting that T2D may induce a more unfavourable cardiovascular risk profile among women. Diabetic women have significantly higher levels of blood pressure and lipids than men with diabetes [27]. Moreover, findings from different studies conducted in the U.K. and the U.S.A. showed that the greater coronary risk associated with T2D observed in women may reflect a treatment bias that favours men. In particular, in these countries, diabetic men with cardiovascular problems are more frequently treated with hypoglycaemic drugs, aspirin, statins or anti-hypertensive drugs than women with similar pathological conditions [28–30].

Moreover, women over 65 years of age have a higher frequency of insulin resistance, dyslipidaemias, central adiposity and hypertension (named the metabolic syndrome) which in turn is a greater risk factor for CVD in women [31,32]. In particular, central adiposity tends to be more pronounced in post-menopausal women than in men playing a determinant role in the increase in CVD risk. It has been demonstrated that visceral adipose tissue contributes to insulin resistance secreting a variety of inflammatory mediators [such as IL (interleukin)-6, TNF-α (tumour necrosis factor α), leptin and resistin]. Moreover, lipid profile [HDL- (high-density lipoprotein) and LDL (low-density lipoprotein)-cholesterol as well as triacylglycerols] dramatically worsens after the menopause favouring atherogenesis [32].

### Cancer

Cancer mortality rates are higher for men than for women in industrialized countries. In some EU countries (i.e. Lithuania, Spain, Latvia, Estonia, the Slovak Republic, Portugal and Croatia), the mortality rates for neoplasms in men are dramatically increased. This gender difference can be explained partly by the greater prevalence of risk factors among men as well as by reduced availability/use of screening programmes for cancers affecting men, leading to lower survival rates after diagnosis [10]. For instance, lung cancer accounts for the greatest number of cancer deaths among men in almost all EU states. In 2011, death rates from lung cancer among men were the highest in all EU countries where smoking habits among men remain very frequent (Hungary, Poland and Croatia). However, lung cancer mortality in American women has increased from 1950 to 1995 by 500% [18]. Evidence has suggested that the development of lung cancer is different in women in comparison with men. Non-smoking women have a 2.5-fold higher risk than men to develop lung cancer at a younger age, but they respond better to treatment. Women who smoke have a higher susceptibility to cigarette-smoking damage probably related to the polymorphism of Glutathione S-transferase (GST) Mu 1, which plays a role in detoxifying environmental carcinogens [33]. However, women with lung cancer survive longer than men, regardless of therapy and stage.

CRC (colorectal cancer) is the second leading cause of cancer death in both genders; in women it occurs 5 years later than in men. For this reason, the population screening for CRC should be extended beyond 70 years of age. Moreover, CRC in women is more often located in the right colon, the histology is mucinous, occult blood in stool may be negative until the last stages and it is frequently diagnosed in an urgent/emergency situation. Nevertheless, the survival is better in female patients with respect to male patients [34]. CRC in women more frequently expresses microsatellite instability showing a lower sensitivity to fluoropyrimidines, cornerstone drugs for the treatment of colorectal carcinoma [35].

Currently, prostate cancer has become the most common cancer among men after skin cancer in the majority of EU countries, particularly among men aged 65 years and over. However, death rates for prostate cancer are lower than for lung cancer. The primary risk factors are obesity, lack of exercise, age and family history [10].

Breast cancer is the second most common form of cancer in women after skin cancer in all EU countries. It can occur in both men and women, but it is very rare in men (10%). The incidence rates of breast cancer have increased in the last decade, but the death rates have diminished or remained stable, indicating an improving of survival rates due to earlier diagnosis and better treatment [36]. Numerous risk factors for breast cancer in women have been identified, including age, personal history of certain benign breast diseases or breast cancer, early menstruation or late menopause, never having been pregnant or having a first pregnancy after age 30, use of oral contraceptives, family history of breast cancer, presence of certain genetic mutations (*BRCA1* and *BRCA2*), history of radiation therapy to the chest, long-term use of combined hormone therapy, use of DES (diethylstilbestrol), increased breast density, alcohol use and obesity after menopause. Risk factors for men for breast cancer include obesity, Klinefelter's syndrome and an excess of breast tissue (http://www.cancer.gov/research/progress/snapshots/breast).

### Neurodegenerative diseases

Women are more affected than men by dementia (definition comprising different conditions including AD and vascular dementia) showing a more frequent and rapid decline of cognitive function with aging. Prevalence rates among populations vary considerably because of methodological reasons (diagnostic criteria, sampling strategies and statistical analysis) [38]. Among people aged 90 years and over, the gender gap rises to 30% of prevalence for men and 47% for women [10].

The biological basis of gender impact on AD and neurodegeneration are still unclear. Indeed, the development and functioning of the central nervous system is strongly influenced by gender. The main risk factor for AD is age, and the fact that the majority of AD patients are females has been attributed to longer life expectancy. However, women are reported to have higher rates of AD than men, even after adjusting for survival [39,40]. The negative effect of the APOE (apolipoprotein E) ε4 allele, one of the most established genetic risk factors for AD, may explain, at least in part, this gender gap. Different studies have observed that female APOE ε4 carriers show a higher risk of AD compared with males [41,42]. A recent paper demonstrated that female APOE ε4 carriers presented widespread brain hypometabolism and cortical thinning compared with female non-carriers, whereas male APOE ε4 carriers showed only a small cluster of hypometabolism and regions of cortical thickening compared with male non-carriers, suggesting that the impact of APOE ε4 on brain metabolism and structure is strongly dependent on gender [43].

AD can be caused by defects in mitochondrial oxidative phosphorylation. Given that the mitochondrial genome (mtDNA) codes for polypeptides that are essential components of the respiratory chain, a number of studies have investigated the association between mtDNA-inherited variants and AD. In particular, research conducted on AD patients and controls from Italy has identified the sub-haplogroup H5 as a risk factor for AD for females in particular and independently of the APOE genotype [44].

It is also worth noting that sex hormones have a critical role in neurodegeneration processes. Oestrogen has been shown to be protective towards AD reducing amyloid β-peptide aggregation and improving neural functions [45–47]. During aging, the decrease in gonadal hormones production is gradual in men (testosterone), whereas in women, the fall of oestrogen is quick after menopause when the incidence of AD suddenly increases [39,48].

A neuroprotective effect of oestrogen on the risk of PD (Parkinson's disease) onset and disease progression has also been reported. Both the prevalence and the incidence of the PD is higher in men than in women [49,50]. In women, the risk of PD is related to the fertile lifespan considering that a later age at menopause is associated with a later age at onset of PD [51,52], whereas a premature menopause increases the risk of PD [53]. These data suggest a relationship between the duration of endogenous oestrogen exposure and the susceptibility to develop PD in women.

### Disability

It is important to underline that women pay for their survival advantage with a worse quality of life in their old age due to an increased prevalence of a variety of disabling non-lethal pathological conditions [15].

Diseases influencing the ADL (Activities of Daily Living) and IADL (Instrumental Activities of Daily Living) scales in women are the consequences of CVD, osteoarthritis, osteoporosis and cognitive decline. Women are more medicalized in terms of frequency of medical visits, days of hospitalization and number of drugs routinely administrated [13,54]. A recent Italian study on a cohort of hospitalized elderly patients (REPOSI) describes a gender dimorphism in the demographic and morbidity profiles as well as in the overall medication pattern of hospitalized elderly people [55,56]. In all EU countries, women reported a poorer self-perceived health, more long-standing illnesses and/or more health problems than men [10]. A possible explanation for this gender-associated health–survival paradox may be found in a higher female sensibility to physical discomfort that led the women to seek medical attention more frequently. Actually, the higher prevalence and severity of arthritis and musculoskeletal disease among older women widely contributes to their worse health and functional status. In particular, women are more frequently affected by severe forms of osteoarthritis affecting the hand, foot and knee, and the incidence of this condition highly increases at the time of menopause, suggesting a role for oestrogens in the pathogenesis of osteoarthritis. Moreover, gender disparities may also be caused by differences in bone strength, posture, ligament laxity, pregnancy and neuromuscular strength [57,58].

### Stress and spousal bereavement

Owing to their longer life expectancy and the tendency to marry older men, women are more likely to become widows. Conjugal loss in advanced age is a stressful life experience able to drastically alter the social environment of the surviving spouse. Therefore widowhood is often associated with a feeling of loneliness, depression, loss of physical and cognitive functions, and poor nutritional status [59–62]. However, spousal bereavement may not have the same implications for women and men. For example, widows maintain higher levels of social contacts with family, friends and neighbours [63] than widowers and this behavioural difference may alleviate some of the undesirable effects of widowhood. Thus, even if widows are more numerous than widowers, it has been shown that widowhood has a more negative impact on health status and mortality in men than women [62,64–66].

## GENDER AND THE IMMUNE SYSTEM

Much research has been carried out into the role of sex hormones in determining lifespan [67] and one hypothesis is that sex hormones appear to influence the immune system. This can determine a sexual dimorphism in the immune response in humans [68]. For instance, females produce more vigorous cellular and humoral immune reactions and are more resistant to certain infections. In contrast, men are more susceptible to many illness caused by viruses, bacteria, parasites and fungi. It is well known that oestrogens, androgens and progesterone affect cells of the innate and adaptive immune system differently during the reproductive phase of life [69]. Oestrogens inhibit NK (natural killer) cell cytotoxicity, reduce neutrophil chemotaxis and consequently inflammation [70,71]. Moreover, macrophages treated *in vitro* with oestradiol display a reduced production of pro-inflammatory cytokines, i.e. IL-1β, IL-6 and TNF-α [72]. Oestrogens and androgens are responsible for a reduced immature number of T-lymphocytes and thymus involution after puberty [73] and can also influence the adaptive immunity in an opposing way. Androgens polarize naïve CD4^+^ T-cells towards the Th1 subset and activate CD8^+^ T-cells; conversely, oestrogens stimulate Th2 responses and activate antibody production [74]. Testosterone increases IL-10 production, and men with androgen deficiencies have higher levels of IL-1β, IL-2 and TNF-α, higher antibody titres and higher CD4^+^/CD8^+^ T-cell ratios [75]. Oestradiol reduces the apoptosis of immature B-cells and also increases somatic hypermutation and isotype-switch recombination leading to high-affinity Ig-producing cells. These effects might contribute to an improved humoral response in women, but also favour the appearance of autoreactive clones and the susceptibility to autoimmune diseases [76]. Moreover, oestrogens down-regulate autoimmune regulator gene (*AIRE*) expression in mTECs (medullary thymic epithelial cells), that plays an important role in protection against autoimmunity, triggering the negative selection of self-reactive T-cells [77]. In addition AIRE induces Treg (regulatory T-cell) development; consequently oestrogens contribute to increased susceptibility to autoimmunity [77]. Several studies showed that females are 2–10-fold more susceptible than males to a series of disabling autoimmune diseases such as rheumatoid arthritis, multiple sclerosis, systemic rheumatoid arthritis, systemic lupus erythaematosus, myasthenia gravis, Sjogren's syndrome and Hashimoto's thyroiditis [78–80]. The better immune response of females is also evident after vaccinations; women reveal higher levels of immunoglobulins and seroconversion and lower rates of disease [75]. In short, sex hormones have different effects on immune responses, with oestrogens exerting an immune-improving action, and progesterone and testosterone having an immune-suppressive effect.

**Figure 1:**
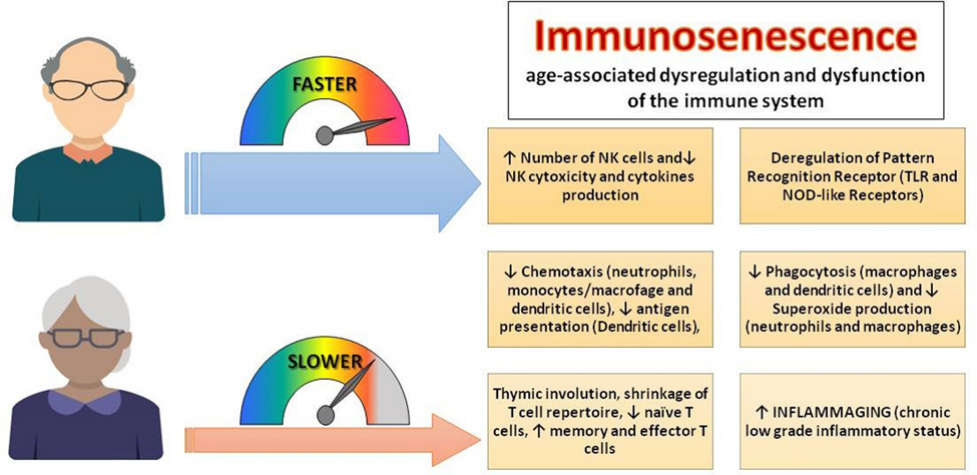
The progression to immunosenescence characterized by age-related changes in immune cells and inflammatory mediators is faster in men than in women [86–93] TLR, Toll-like receptor.

The sudden loss of ovarian oestrogen and progesterone production that characterizes menopause, induces pathophysiological changes in different organs and systems [81]. Menopause reflects the inevitable final hallmark of a woman's fertile lifespan and of the above-described beneficial effects of oestrogens on immune responses. Menopause affects various women's health aspects, including bone density, breast cellular composition, cardiovascular health, mood/cognitive function and sexual wellbeing. Moreover, old women lose their immunological privilege towards infection [69] because the rapid reduction of oestrogen levels results in an increased susceptibility and mortality towards a series of infectious diseases (hepatitis, and meningococcal and pneumococcal infections) [69,79]. It is noteworthy that women will soon spend half of their life in post-menopause, if the current trend of increasing human life expectancy should persist. Various studies have reported an association between late-onset menopause and reduction in all causes of morbidity and mortality [82]. Both fecundity at an older age and a high age at menopause have been associated with longevity [83]. Several studies have suggested that ovarian sex steroid loss favours immunosenescence by contributing to the remodelling of the immune system. Immunosenescence is a multifaceted phenomenon that increases morbidity and mortality due to infections and age-related pathologies, and is characterized by changes in innate and adaptive immune responses to foreign antigens [84,85]. In Figure 1, the main aspects of immunosenescence [86–92] are shown and it is indicated that age-related changes in immune cells and inflammatory mediators, i.e. the progression to immunosenescence, are faster in men than in women [93].

Functional aspects of age/gender-specific differences of the immune system and its interplay with changing sex steroid hormone levels have not been investigated extensively. Post-menopausal women exhibit a reduced number of total lymphocytes, mainly B- and CD4^+^ T-lymphocytes [94] and an altered expression of inflammatory mediators such as an increased plasma level of IL-1β, IL-6, IL-10 and TNF-α [95–98]. After a transient rise in post-menopausal women, IFN-ɣ (interferon γ) levels gradually decrease with age. Yet the production of IL-10 increases during the post-menopausal period [95]. Moreover, in *in vitro* stimulation studies, IFN-ɣ and IL-17 secretion is diminished in aged men in comparison with women [99]. In contrast, the anti-inflammatory cytokine IL-10 increases in aged women but not in men. Centenarians, mainly females, present markers of inflammation [e.g. increased plasma levels of IL-6 and CRP (C-reactive protein) and hypercoagulable state], but do not suffer most of the detrimental effects of inflammaging. Accordingly, centenarians seem to be equipped with gene variants that allow them to optimize the balance between pro- and anti-inflammatory molecules, thus minimizing the effects of the lifelong exposure to environmental insults and stressors [100].

## GENDER AND NUTRIENT-SENSING PATHWAYS

DR (dietary restriction) without malnutrition, intended as a reduced intake of all dietary constituents except vitamins and minerals, is a well-known intervention to improve most aspects of health during aging and to extend lifespan in model organisms from invertebrates and rodents to primates, including humans [101]. However, in humans, this practice remains difficult, if not impossible, to sustain because it envisages unrealistic levels of self-deprivation, can impair reproductive function and libido, resistance to infection and wound healing, and can increase the risk of osteoporosis and fractures, anaemia and cardiac arrhythmias [101]. Therefore interest in interventions able to recapture the beneficial effects of DR has grown. Among the mechanisms mediating the effects of DR, particular attention has to be paid to nutrient-sensing pathways, such as IIS [insulin and IGF-1 (insulin-like growth factor 1) signalling] by their transcription factor FOXO (forkhead box O) or via mTOR (mammalian target of rapamycin), which are considered key modulators of lifespan and the aging process [102,103]. These highly conserved pathways are designated to couple nutritional status to energetically expensive processes, such as growth, reproduction and metabolism [104]. Several studies on experimental animal models have tried to disentangle the effect of IIS/TOR (target of rapamycin) signalling network on biological processes. Specifically, interventions aimed at the down-regulation of this pathway affect the expression of hundreds of genes involved in immunity and stress responses, activate anti-aging responses and are able to extend lifespan mimicking the action of DR.

On the whole, data on animal models have shown that genetic mutations inhibiting IIS and TOR nutrient sensing signalling have a stronger effects on lifespan extension in females [101]. For example, *Drosophila* mutants with impaired insulin-like signalling have a significant life extension in females [105,106] and heterozygous IGF-1R (IGF-1 receptor)-knockout female mice are long-lived and show a higher oxidative stress resistance than wild-type mice, whereas the difference is not significant in males [107]. The deletion of S6K1 (ribosomal S6 protein kinase 1), a component of the nutrient-responsive mTOR signalling pathway, leads to a significantly increased lifespan and to an improvement in a number of age-sensitive biomarkers of aging (fewer memory and more naïve T-cells, lower plasma leptin levels and fat mass) in females only [108]. Similarly, rapamycin, an inhibitor of mTOR kinase, increases the lifespan of genetically heterogeneous UM-HET3 mice more in females than in males at each dose evaluated [109].

In humans, IIS and mTOR signalling have been investigated for their role in the development of diseases, such as diabetes and cancer, and for their impact on longevity [110]. Large cohort studies have shown a significant interaction with gender. For example, genetic variation in IIS pathway components [GHRHR (growth hormone-releasing hormone receptor), GH (growth hormone), IGF-1, insulin, IRS1 (insulin receptor substrate 1)] have a higher influence on body size and are more beneficial for old age survival in women with respect to men [111]. Some human studies have investigated the role of sex hormones in regulating the somatotropic axis (GH and IGF-1) underlining gender differences in the impact of suppression of the nutrient-sensing pathways on aging and longevity. For instance, oestradiol reduces hepatic sensitivity to GH, whereas testosterone plays an opposite role enhancing the growth-promoting effects of the somatotropic axis [13,112,113] and increasing the risk of some age-related pathologies such as prostate cancer and cardiomyocyte hypertrophy [114,115].

To date, there is a lack of data on the effect of DR on human longevity and whether this practice has a different impact according to the gender is largely unknown. However, increasing interest has been paid to trials on the effects of IF (intermittent fasting) or adjusted rhythm of feeding on women's health. A study in overweight or obese pre-menopausal women has demonstrated that IF (two non-consecutive days per week over a 6-month period) is an effective intervention to reduce weight, fat mass and waist circumference as well as to improve insulin sensitivity and other biomarkers such as total and LDL-cholesterol, triacylglycerols, CRP and arterial blood pressure [116]. Therefore IF may be considered as an alternative and more feasible practice than DR to reduce disease risk [116]. Interestingly, a ‘breakfast diet’ (980 kcal breakfast, 640 kcal lunch and 190 kcal dinner; 1 kcal=4.184 kJ) on lean women with polycystic ovary syndrome improves glucose metabolism, decreases free testosterone and increases the ovulation rate with respect to an isocaloric ‘dinner diet’ (190 kcal breakfast, 640 kcal lunch and 980 kcal dinner) [117].

However, to date, few studies have assessed the differences between men and women in response to nutritional interventions. Several papers have described the effects of a 4-week fully controlled isoenergetic Mediterranean diet on a group of 38 men and 32 pre-menopausal women (24–53 years). The results have shown an improvement in lipid profile, cardiovascular risk and inflammation markers which was significant in both genders [118,119]. Such a short-term consumption of Mediterranean diet significantly ameliorates insulin homoeostasis [120], leads to a favourable redistribution of LDL subclasses [121] and reduces adiponectin levels [122] only in men. The greater improvements in dietary intakes obtained in men with respect to women can explain, at least in part, these gender-related responses [123], but it is worth considering that gender differences in the remodelling, distribution and secretory activity of adipose tissue as well as the levels and ratio of androgenic and oestrogenic steroids may play a fundamental role in metabolism homoeostasis. These data underline the importance of considering gender in further studies evaluating the effects of dietary intervention on diseases, aging and longevity taking into account that men and women can show very different responses and require personalized treatments.

## HUMAN POPULATION MODELS TO STUDY GENDER EFFECT ON AGING AND LONGEVITY

The particular combination of genetic, environmental, historical, anthropological, socio-economic and cultural factors as well as geographical origin could contribute to the longer female life expectancy worldwide. To increase our knowledge on these aspects, the model of centenarians could represent a useful approach. These extraordinary individuals (mostly women) are characterized by a peculiar and heterogeneous phenotype embodying the best example of longevity and successful aging. Most of them have survived, escaped or delayed the onset of major age-related diseases [124–126]. Centenarians are the outcome of a number of biological processes that exert their effects lifelong, from birth (and even before) until the extreme limits of human life. From a demographic point of view, the high number of centenarians in our societies is the integrated result of complex interactions between humans and their environment(s) which underwent consistent changes since the beginning of the 20^th^ century and which are continuing today. Therefore the study of centenarians represents a sort of ‘historical probing’ that allows the tracing of the above-mentioned complex basis of the longevity today. A historical perspective of demographic data on gender and longevity in Italy is shown in Box 1.

The model of centenarians has some disadvantages due to their rarity, lack of an age-matched control group and phenotypical frailty related to their extreme age. Literature suggests that longevity ‘runs in families’ through different generations [136] and, indeed, centenarian offspring appear to be healthier [137,138] and to have a more favourable biological signature [139] with respect to age-matched controls, thus representing a suitable model to identify early biological factors/markers correlated to healthy aging and higher ‘risk’ of longevity. Thus female offspring of centenarian parents could represent a peculiar subgroup of women characterized by a survival advantage not accompanied by the worst quality of life typical of elderly women.

Within this scenario, where plenty of data have described the hormonal, immunological and metabolic gender differences, the study of long-lived families has allowed us to address peculiar aspects of the genetics of aging in women, following the ‘three genetics conceptualization’ we have proposed recently [140]. We have suggested that an integrated investigation of nuclear genetics, mitochondrial DNA genetics and gut microbiome is essential to grasp the genetic contribution to aging and longevity in humans considered as meta-organisms.

Box 1 **Demographic data on gender and longevity: a historical perspective in Italy**

Low-mortality countries, as well as Italy, in the recent decades have seen a process of reduction in mortality at all ages of life that has allowed pronounced gains in life expectancy [127]. Currently, the average life expectancy at birth in Italy is among the highest in the world, having reached 80 years for men and 85 years for women. The improvement in living conditions, education, nutrition and lifestyles, and progress in the prevention, diagnosis and treatment of diseases, have been crucial in reducing the risk of death even in advanced ages of life. Indeed, mortality rates at older ages showed a linear downward trend between 1950 and 2012 [128].

The decline in old-age mortality is thought to be the main cause of the dramatic increase in centenarians [129], whose number doubles approximately every 7 years. According to data of the Italian National Institute of Statistics (http://demo.istat.it/), the number of centenarians has reached 19095 (i.e. over 31 per 100 thousand residents) on 1 January 2015. At the same date, according to the ISTAT register, 878 residents on Italian territory were semi-supercentenarians (persons aged 105 or over), whereas 17 were supercentenarians (persons aged 110 or over).

Increased levels of survival are linked to the long process of epidemiologic transition that saw a radical transformation of mortality in its gender, age and cause profiles [131,132].

**Figure 2:**
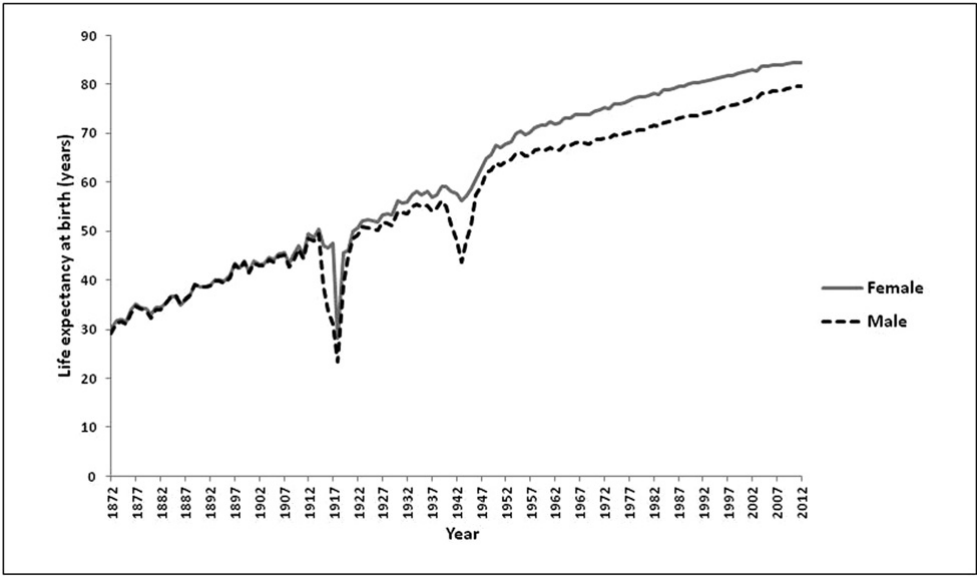
Evolution of life expectancy at birth in Italy (1872–2012) Data from the Human Mortality Database (University of California, Berkeley, and Max Planck Institute for Demographic Research, http://www.mortality.org).

As shown in Figure 2, life expectancy in Italy was very similar for males and females until the early 20th Century. Afterwards, the evolution of life expectancy, while being dramatically marked by a sharp drop during the two World Wars, showed a differentiation between males and females that reached a maximum of 6.75 years in 1979 and 1980.

The recent decrease in the gender gap is mainly due to the reduction of excess male mortality between the ages of 45 and 75 years. On the other hand, the disadvantage of men compared with women at older ages is emphasized. This phenomenon might be related to a generation effect: whereas in the younger generations with more healthy lifestyles the gap is reducing, the cohorts born in the early 20th Century and the mid-1930s are ‘carriers’ of an excess mortality [133]. The current centenarians emerge from these cohorts and show geographical differences in the female/male ratio (F/M), which is higher in the North-West and North-East areas (around 7:1 and 6:1 respectively), intermediate in the Centre (around 5:1) and lower in the South and Islands (around 4:1), according to the most recent ISTAT and census data. It is worth mentioning that in a mountainous zone of Sardinia (Nuoro province), an exceptionally high number of male centenarians was identified, together with an unusually low F/M ratio [134].

The pattern of distribution of extremely long-lived individuals is certainly affected by environmental factors which shaped the geography of life expectancy in Italy through a different impact on the main causes of death in the elderly [135]. However, a role of genetic factors is suggested by the finding of a correlation between centenarians' gender ratio across the national territory and the first principal component obtained by studying the polymorphic variation at 95 different loci [5].

### X chromosome inactivation (XCI) skewing in human aging and longevity

In cells from females, one of the two X chromosomes is epigenetically and randomly inactivated in early embryonic life. Young women are a mosaic of two cell populations in which either the maternal or the paternal X chromosome is inactivated, and the ratio is close to 50% for each chromosome. A general concordance was seen in the XCI (X chromosome inactivation) pattern between haemopoietic tissue (blood and/or spleen) and several other tissues (e.g. brain, skin, heart, lung, muscle, kidney and gastrointestinal). According to the ‘Heterogametic Sex Hypothesis’, having two copies of the X chromosome may be advantageous for females because of possible selection with age of the better X chromosome while inactivating the deleterious one [141]. In addition, previous data reveal that a small portion (∼17%) of the genes on the inactivated X chromosome are partially active providing a further survival advantages for females [141]. During aging, a marked deviation from the equivalent ratio (50:50) between maternal and paternal X chromosome inactivation occurs (skewing of XCI) in blood cells and the concordance of XCI among tissues may weaken with age. In particular, comparing haemopoietic tissues and brain in the oldest women, the greatest difference between inactivation values of the two tissues were found [142]. The XCI patterns in brain are of particular clinical relevance, because the X chromosome is relatively enriched for genes involved in neuronal functioning [143]. Some authors suggested that age-associated XCI skewing could be involved in the pathogenesis of several diseases such as autoimmunity and cancer [144]. Our proposed experimental model of longevity/healthy aging consisting of female centenarians, their female offspring, female offspring born from non-long-lived parents (age-matched controls) and young women has allowed us to extend to centenarians the study of XCI skewing and to demonstrate that this process was significantly less severe and frequent in centenarian offspring compared with their age-matched controls [145]. These results highlight a possible detrimental link between the rate of XCI skewing and healthy aging/longevity, fitting the hypothesis that the balanced female mosaic is a winning strategy, sustaining a co-operative adaptive mechanism with possible biological advantages, whereas a skewed situation in favour of one of the two X chromosomes would represent an unfavourable condition to attain health and longevity. Conversely, the absence of a similar mosaic strategy in men might contribute to their shorter lifespan [1].

A recent paper has described the correlation between SEMs (stochastic epigenetic mutations) (i.e. rare or stochastic epimutations not shared among individuals) and XCI skewing during aging demonstrating that the number of SEMs was low in childhood and increased exponentially with age [146]. Moreover, a multivariate analysis has indicated a significant correlation between SEMs and degree of XCI skewing after adjustment for age, indicating for the first time that XCI skewing may not be a direct consequence of aging, but is mediated by the number of SEMs. The data from this study support the hypothesis that an increased number of SEMs might influence haemopoietic stem cells viability or might create conditions able to induce clonal stochastic loss of a specific type of haemopoietic cells [146].

### mtDNA and gender in human aging and longevity

Mitochondria are considered to be important determinants of cell aging because they are involved in several fundamental processes such as cellular energy/ATP production, the urea cycle, heat production, apoptosis, inflammasome activation and cell senescence. Mitochondria are also the main producers of ROS (reactive oxygen species), the most important by-products of OXPHOS (oxidative phosphorylation), which, besides their physiological role in cell signalling, have been suggested to play a role in the aging process as well as in age-related diseases. Data from primary culture of fibroblasts from long-living individuals, including female centenarians, indicate that longevity is characterized by a preserved bioenergetics function probably attained by a successful mitochondria remodelling that can compensate for functional defects through an increase in mass, i.e. a sort of mitochondrial ‘hypertrophy’ [147].

Another aspect deserving particular attention in the study of female longevity is the complex and contradictory role of mtDNA variability. mtDNA is an active part of the genetic machinery of each cell and has an active cross-talk with the nuclear genome. Despite its limited length (16569 bp), the mtDNA encodes few genes with a quantitatively relevant action because of the high copy number of mtDNA in each cell. mtDNA is inherited only through the mother and its germline variants (haplogroups), and D-loop mutations were found to be associated with longevity in several populations indicating a maternal component of longevity.

In particular, the EU large project GEHA (Genetics of Healthy Ageing) studied 2200 ultra-nonagenarians (90+) from different EU countries belonging to 90+ sibpairs together with the same number of sex- and geographically-matched younger controls, and was able to identify different haplogroups related to longevity in males and females. The J2 haplogroup was associated with male longevity, whereas the H2 and T2 haplogroups were associated with female longevity [148]. Taking advantage of the complete sequencing of a high number of mtDNA molecules, it was also possible to evaluate for the first time the cumulative effect of specific and concomitant mtDNA mutations, including those that have a low or very low impact. The analysis of the mutations occurring in different OXPHOS complexes showed a complex scenario with a different mutation burden in nonagenarian persons compared with controls. In particular, mutations in subunits of OXPHOS complex I had a beneficial effect on longevity, whereas the simultaneous presence of mutations in complex I and III and in complex I and V seemed to be detrimental [148]. The final conclusion was that “particular rare mtDNA mutations present only in specific populations might be beneficial (or detrimental) for longevity and may explain part of the genetic component of longevity in that population, similarly to what has been suggested for private nuclear DNA polymorphisms” [148].

mtDNA mutations are transmitted from centenarian mother to the progeny. One of the factors that can contribute to aging and longevity is the accumulation with age of mtDNA mutations. mtDNA heteroplasmy, i.e. the presence in the same cell of wild-type and mutated mtDNA molecules, has been supposed to have a double role, fuelling mitochondrial dysfunction and, at the same time, functioning as a reservoir of genetic variability helping the cells to cope with environmental and physiological stressors during life [149,150]. To test the hypothesis that mtDNA heteroplasmy could play a role in human aging and longevity, Giuliani et al. [151] exploited two approaches: (i) the previously described informative model, i.e. 31 centenarian families constituted by the centenarian mother plus the female offspring, in comparison with 28 female offspring of not long-lived parents; (ii) the most recent technology of ultra-deep mtDNA sequencing (average coverage of 49334-fold for each 853bp mtDNA fragment examined). This method allowed the detection of 119 heteroplasmic positions with a minor allele frequency ≥0.2%. The results indicate that a low level of heteroplasmies are transmitted and maintained within families until extreme age. However, a non-heteroplasmic variant associated with longevity and healthy aging was identified but a particular and unique heteroplasmy profile for each family was drawn. Therefore mtDNA heteroplasmy appears to be a familial trait transmitted by the mothers which can contribute to healthy aging and longevity [151].

On the other hand, a number of studies have investigated the association between mtDNA inherited variants and multifactorial diseases, such as diabetes [152], ischaemic disease [153] and neurodegenerative diseases such as PD [154] and AD [44]. As described previously, a high-resolution analysis (sequencing of displacement loop and restriction analysis of specific markers in the coding region of mtDNA) found that sub-haplogroup H5 is a risk factor for AD in particular for females and independently of the APOE genotype partially explaining the higher prevalence of AD in women [44].

### Gut microbiota and gender in human aging and longevity

Humans have to be considered as metaorganisms due to symbiotic relationship with the numerous microbial communities (‘microbiota’) present in various anatomical locations of the human body. Several hundreds of individual bacterial species colonize mouth, upper airways, skin, vagina and intestinal tract constituting a complex and dynamic ecosystem which cross-talk with the environment as well as the rest of the body, including liver and brain among others. At present, the microbiota associated with the intestinal tract [GM (gut microbiota)] is the most studied. The GM are essential for the synthesis of some fundamental nutrients and energy production from food and are able to strongly modulate innate and specific immunity. The gastrointestinal tract of newborns becomes colonized immediately after birth with micro-organisms, mainly from the mother. The composition of vaginal tract microbiota of the mother, the mode of delivery (natural or Caesarean) and breast or formula feeding have a deep impact on the GM of human offspring since the very beginning of life. Strong evidence has suggested that the early composition of the microbiota of newborns plays an important role for the postnatal development and functionality of the immune system [155].

Data regarding the association between genders and specific GM communities are still unreliable even if some reports found that some specific taxa (*Bacteroides*, *Ruminococcus*, *Eubacterium* and *Blautia*) are more abundant in men, whereas *Treponema* is prevalent in women [78,156]. Probably, these differences in GM composition are due to lifestyle and dietary factors as well as cultural gender-related habits rather than sex hormone effects [78]. Alterations of the GM have been observed in numerous diseases such as obesity, T2D, inflammatory bowel disease and CRC. In particular, specific signatures of GM patterns are associated with autoimmunity affecting prevalently women and contributing to the increase in their morbidity [78]. Thus there is an urgent need to consider the role of gender background in the GM ecology and its relationship with autoimmunity disease onset and therapy effects. This consideration is reinforced by the fact that the importance of GM in human aging is dramatically emerging. This endogenous ecosystem, together with the external antigenic load, is coming out as a crucial driving force of the homoeostasis of the immune system, and lifelong GM changes, from newborns to centenarians, can represent an important source of inflammatory stimuli. Our group has shown that female centenarians have a different composition of the GM in comparison with sex-matched younger persons, which is associated with an increase in inflammaging (high plasma levels of pro-inflammatory cytokines such as IL-6 and IL-8). In general, with aging, a decrease in the biodiversity of the composition of the GM is observed, with a trend towards an increase in potentially pathogenic bacteria (pathobionts) with respect to the beneficial ones (symbionts producing butyrate and other short-chain fatty acids) [157]. However, data on the remodelling of the GM and its association with inflammaging are still lacking in men, underlining again the importance of conducting gender-specific studies to fill this gap.

## AN AGENDA FOR THE FUTURE: A MANDATORY NEED FOR A GENDER-SPECIFIC MEDICINE

The aging process starts ‘*in utero*’ and early events exert potent effects later in life both in adult age and in old age. This lifelong perspective of aging and age-related diseases let emerge the importance of going beyond sex and to consider ‘gender’. Indeed, men and women differ not only biologically (biology, physiology and genetics), but also regarding lifestyle and habits (smoking, nutrition, physical activity, type of work and education, among others) as well as regarding the capability of coping with stress (spousal bereavement, serving as care-givers to family members). These biological and non-biological factors interact continuously lifelong, playing an overwhelming role in modulating health and/or the propensity to diseases and disabilities later in life.

From basic to clinical sciences, there is a mandatory need for studies where gender is appropriately and fully considered. The enormous progress of medicine in the last 50 years has been reached by scientific investigations and publications where gender has been rather neglected: ‘put gender on the agenda’ has been repeatedly stated by top journals such as *Nature* since 2010 [158,159].

Gender medicine can be considered quite a new but mandatory dimension of medicine that has to go much deeper in understanding the differences between men and women regarding all pathophysiological pathways, clinical characteristics and pharmacological responsiveness, as well as the importance of lifestyle and cultural aspects [18].

Within this scenario, it is even better to speak about a ‘gender-specific medicine’ and not only an indefinite and/or separated ‘gender medicine’ since the gender perspective is broader, should be more pervasive and penetrate all specialties of medicine. Gender medicine is not a separate exercise, or a separate branch of medicine. Therefore gender should be the focus for the clinical approach and this task requires a deep cultural change of mind as well as a reorganization of clinical services in all countries and health systems. Gender medicine is even more necessary in neglected countries such as in Africa where the conditions of women are worse and gender differences are stronger and have a higher impact on the health status. At the same time, it is currently no longer possible to conduct medical as well as biological sciences and education programmes without taking into considerations gender differences in the medical schools as well as in all educational programmes.

The knowledge of the biology of gender differences in humans are still in their infancy and there is an urgent need for specifically targeted large studies across countries, to take into account the above-mentioned cultural and anthropological differences in a globalized world where migration of persons from countries characterized by different genetic, cultural and anthropological traits and habits is a hot topic.

In conclusion, the development of a gender-specific medicine is of the utmost importance in order to complete our understanding of the main mechanisms of aging as well as the differences in prevention, care, treatment, evolution and outcomes of non-communicable diseases in both genders.

## Abbreviations

AD: Alzheimer's disease
AIRE: autoimmune regulator
APOE: apolipoprotein E
CRC: colorectal cancer
CRP: C-reactive protein
CVD: cardiovascular disease
DR: dietary restriction
GH: growth hormone
GM: gut microbiota
GST: Glutathione S-transferase
HF: heart failure
IF: intermittent fasting
IFN-γ: interferon γ
IGF-1: insulin-like growth factor 1
IHD: ischaemic heart disease
IIS: insulin and IGF-1 signalling
IL: interleukin
LDL: low-density lipoprotein
mTOR: mammalian target of rapamycin
NK: natural killer
OXPHOS: oxidative phosphorylation
PD: Parkinson's disease
SEM: stochastic epigenetic mutation
T2D: Type 2 diabetes
TNF-α: tumour necrosis factor α
TOR: target of rapamycin
XCI: X chromosome inactivation
